# Cisplatin-Induced Toxic Optic Neuropathy in a Patient With Hodgkin’s Lymphoma

**DOI:** 10.7759/cureus.69140

**Published:** 2024-09-10

**Authors:** See Yee Bee, Jemaima Che Hamzah, Rona A Nasaruddin, Ainal Adlin Naffi

**Affiliations:** 1 Department of Ophthalmology, Faculty of Medicine, Universiti Kebangsaan Malaysia, Kuala Lumpur, MYS

**Keywords:** cisplatin, hodgkin lymphoma, splinter hemorrhage, swollen optic disc, toxic optic neuropathy

## Abstract

Hodgkin's lymphoma (HL) is commonly treated with multi-agent chemotherapy, with cisplatin being a key component due to its effectiveness in inducing DNA crosslinking and causing cancer cell death. Despite its therapeutic benefits, cisplatin can lead to serious ocular complications, including a rare but severe condition known as cisplatin-induced toxic optic neuropathy. This condition, while uncommon, has the potential to cause significant and irreversible visual impairment, particularly in pediatric patients, even when cisplatin is administered at standard therapeutic doses. The lack of specific guidelines for ocular monitoring during cisplatin therapy exacerbates the challenge of managing this complication, highlighting the importance of monitoring, early detection and intervention.

This case report describes a 16-year-old male pediatric patient with stage IVA HL who was admitted for dexamethasone, high-dose cytarabine, and platinum (DHAP) chemotherapy to treat relapsed disease. He developed sudden, painless bilateral vision loss three days after the discontinuation of the DHAP regimen, which had been stopped due to tumor lysis syndrome and acute kidney failure. The patient presented with severely reduced visual acuity and optic disc swelling in both eyes. Despite receiving high-dose intravenous methylprednisolone, visual improvement was minimal. Unfortunately, the patient did not survive due to disease progression.

This case report emphasizes the critical need for careful ocular monitoring and the prompt involvement of ophthalmologists to manage and prevent severe and irreversible ocular complications associated with cisplatin therapy.

## Introduction

Hodgkin's lymphoma (HL) is a distinctive type of hematological malignancy characterized by the presence of malignant Reed-Sternberg cells within an inflammatory environment [1]. The primary treatment modality for HL involves multi-agent chemotherapy regimens, of which cisplatin is the key component. Dexamethasone, high-dose cytarabine, and cisplatin (DHAP) are employed as a treatment regimen for refractory or relapsed HL and non-HLs, either as a primary or secondary option [2]. Additionally, DHAP is utilized as a mobilization regimen before high-dose therapy, accompanied by peripheral hematopoietic progenitor cell support [3]. Cisplatin exerts its anti-cancer effects by forming DNA crosslinks, leading to cell death [1]. Ocular side effects are commonly observed following the administration of the platinum salts. These effects encompass alterations in macular pigment, leading to changes in central vision, complications in retinal microvasculature resulting in the formation of new blood vessels, vascular retinopathy including central retinal occlusion, and papillitis associated with vasculitis [4].

## Case presentation

A 16-year-old Malay male was diagnosed with stage IVA HL without B symptoms in June 2020. He presented with symptoms of superior vena cava obstruction at his initial presentation. He completed eight cycles of bleomycin, etoposide, doxorubicin hydrochloride, cyclophosphamide, vincristine, procarbazine, and prednisolone (BEACOPP), along with two cycles of involved field radiation therapy, in 2021. However, he experienced a relapse of the disease, as evidenced by a repeated positron emission tomography (PET) scan showing new hypermetabolic disease at multiple sites including the cervical region, mediastinum, spleen, bone marrow, and lung, as well as a mediastinal lymph node biopsy revealing nodular sclerosis classic HL.

Consequently, he was admitted for peripheral blood stem cell (PBSC) mobilization with DHAP chemotherapy due to his relapsed HL. On the fourth day of the first cycle of the DHAP regimen, the patient developed sensorineural hearing loss, tumor lysis syndrome, and acute kidney failure, which resulted in the discontinuation of the treatment. He required ongoing plasma exchange due to tumor lysis syndrome and acute kidney failure, with urea levels of 30.5 mmol/L (normal range: 3.2-7.4 mmol/L) and creatinine levels of 594.9 μmol/L (normal range: 63.6-110.5 μmol/L). During each plasma exchange, he was transfused with fresh frozen plasma and cryoprecipitates to address his severe coagulopathy.

**Figure 1:**
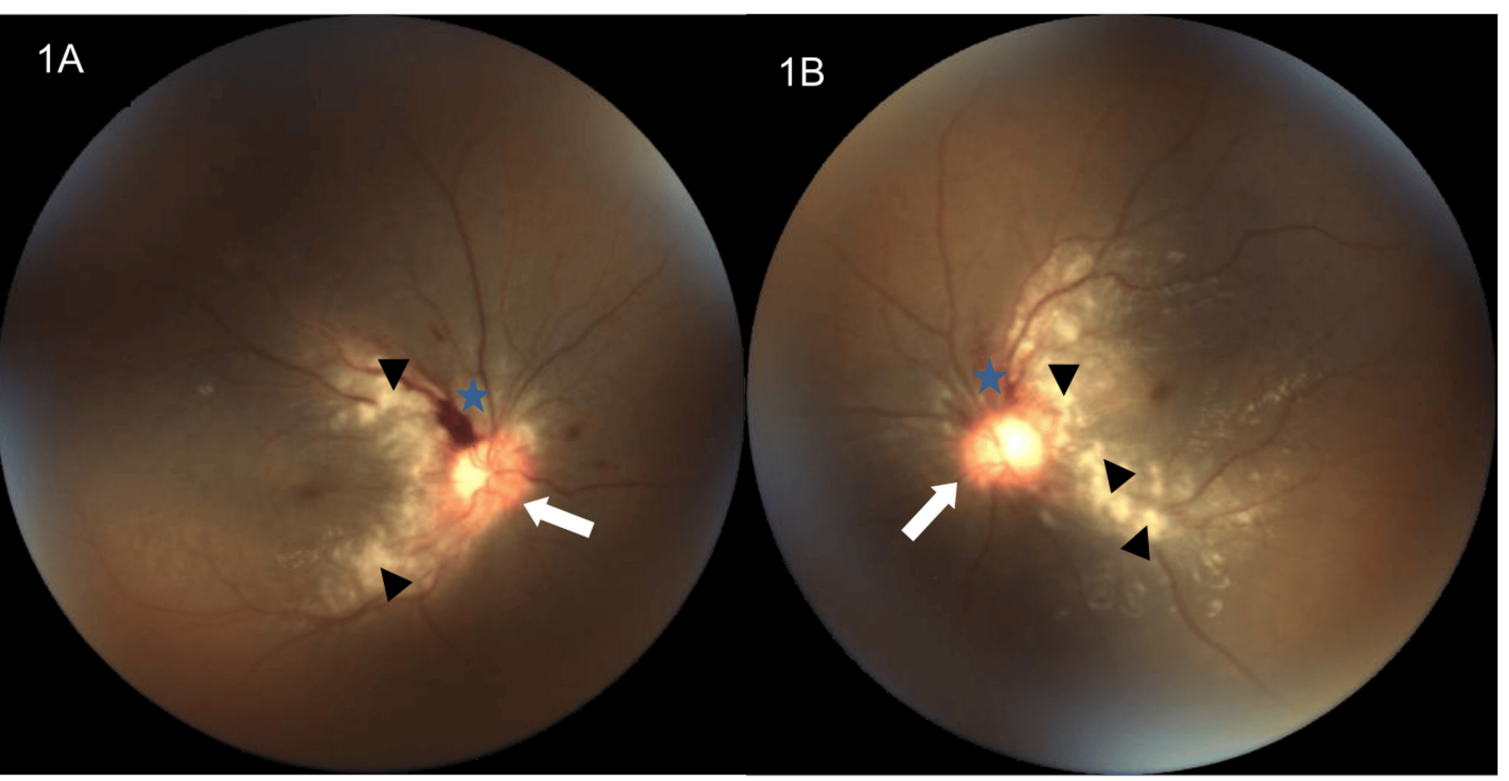
Bilateral fundus photos of the patient's eyes during the first presentation (1A and 1B) Bilateral optic disc swelling (white arrows), splinter hemorrhages (blue stars), and multiple cotton wool spots (black arrowheads) were observed in the posterior pole of the retina.

However, he developed sudden onset of blurred vision bilaterally three days after discontinuation of the DHAP regimen. The total cumulative dose of cisplatin he received was 800 mg/m². He denied floaters, eye pain, or eye redness. Upon examination, he was alert but ill-looking with ongoing plasma exchange on him. Examination revealed visual acuity of 3/60 in the right eye and hand movement perception in the left eye, without relative afferent pupillary defect. However, both pupils were sluggish in direct and consensual light reflexes. The anterior segment of both eyes appeared normal, and intraocular pressure was within the normal range. Fundus examination revealed bilateral optic disc swelling, as evidenced by circumferential blurred optic disc margins with hyperemic discs and splinter hemorrhages, along with multiple cotton wool spots in the posterior pole of the retina (refer to Figures 1A, 1B). Due to the patient being bedridden and clinically unfit, other modalities of examination and ancillary tests were not feasible.

The patient was treated for optic neuritis and received IV methylprednisolone, 250 mg four times a day for three days, with a planned transition to oral prednisolone at 1 mg/kg/day for 11 days. By the third day of IV methylprednisolone treatment, there was a slight improvement in vision in the left eye, with visual acuity improving to counting fingers. After completing a three-day course of IV methylprednisolone, the patient’s Glasgow Coma Scale (GCS) score declined, and he developed neutropenic sepsis along with an upper gastrointestinal bleed. He required intubation and inotropic support to stabilize his condition. An esophagogastroduodenoscopy (OGDS) was conducted, during which a Forrest IIA ulcer was identified and clipped to control the bleeding. Despite treatment, his condition worsened with ongoing coagulopathy and severe metabolic acidosis. Ultimately, the patient succumbed to disease progression and associated complications.

## Discussion

Cisplatin-induced toxic optic neuropathy, including optic neuritis, is a rare adverse effect, particularly in the pediatric population. Most cases are mild and reversible, often going undiagnosed due to the lack of ophthalmologist involvement. The clinical manifestations of platinum-induced toxicity include retrobulbar neuritis, papillitis, neuroretinitis, and serous retinal detachment [5-14], potentially leading to symptoms such as blurred vision or altered color perception.

There are reports in the literature of platinum-induced optic neuropathy. The first case was documented by Caraceni et al. in 1997 [7], describing bilateral optic neuropathy in a patient with ovarian carcinoma who had received high doses of cisplatin (160 mg/m²) and carboplatin (640 mg/m²) [7]. The patient developed bilateral optic neuritis 13 weeks after discontinuing cisplatin, with recovery observed after one year through serial Visual Evoked Potential tests [7]. O'Dea et al. documented a case series of oxaliplatin-induced optic nerve toxicity, where one out of four patients displayed bilateral optic disc edema, although all changes were reversible [8]. In 2009, Fischer et al. [9] reported a patient with serous-papillary ovarian carcinoma who developed carboplatin-induced bilateral optic disc swelling, leading to partially reversible visual impairment after the eighth day of the fourth chemotherapy cycle containing carboplatin [9]. Beaumont et al. also reported a case of bilateral optic neuropathy induced by oxaliplatin [4]. Lewis et al. [10] documented unilateral optic disc swelling following carboplatin chemotherapy for ovarian cancer, while Shihadeh et al. [11] described a case of carboplatin-induced bilateral optic neuropathy resulting in irreversible vision loss in a patient with bladder cancer. Maleki et al. [12] reported carboplatin-induced bilateral optic neuropathy in a patient with metastatic squamous cell carcinoma of the tongue, leading to no light perception in the left eye. Kim et al. [13] described a patient with stage 4 small cell lung cancer and multiple intra-abdominal metastases who developed unilateral optic nerve pallor and bilateral sclerosis and attenuation of arteries one week after the second cycle of chemotherapy containing carboplatin. Hasik et al. [14] conducted a disproportionality analysis, identifying 69 signals of ocular adverse events associated with platinum-based chemotherapy drugs. Among these, choroidal infarction and orbital hemorrhage were notably linked to carboplatin, optic disc hyperemia was associated with oxaliplatin, and cortical blindness with cisplatin [14].

The mechanism behind cisplatin-induced retinal and optic nerve toxicity remains unclear. However, insights from studies on acute neuropathy provide some speculation. In research using genetically mutated mice, authors observed that deficient voltage-dependent sodium channels in various retinal neurons impair photoreceptor function, potentially leading to retinal toxicity through dysfunction of the retinal neural pathway and photoreceptors [15]. Similarly, in optic neuropathy, abnormalities in sodium channels caused by platinum may lead to excessive sodium influx into cells, playing a crucial role in ischemic damage to the optic nerve and subsequent neuronal apoptosis [11,16]. It is important to note that retinopathy signs typically exhibit reversibility, while optic nerve-related conditions tend to be irreversible. Moreover, like other toxic causes, the koniocellular system, responsible for processing yellow-blue opponent fields, is particularly affected.

Our patient presented with bilateral optic disc swelling, indicative of cisplatin-induced toxic optic neuropathy, occurring three days after stopping cisplatin-containing systemic chemotherapy. Additionally, the patient exhibited sensorineural hearing loss, which further suggested cisplatin toxicity. This finding is similar to those reported by the authors [7-13]. However, our report has some limitations. Firstly, the patient's condition prevented further ancillary tests that could have ruled out other differential diagnoses, such as an infiltrative or compressive cause. Secondly, there were no baseline ocular records, such as visual acuity or fundus examination, prior to DHAP chemotherapy, making it impossible to confirm that the vision loss occurred following the therapy. Lastly, visual recovery or outcome could not be documented, as the patient succumbed to disease progression.

Unfortunately, there are no guidelines for monitoring ocular assessment when the patient is initiated on cisplatin chemotherapy. Managing cisplatin-induced toxic optic neuropathy is more challenging than preventing it. Therefore, ensuring prompt referral to an ophthalmologist before commencing cisplatin treatment and conducting daily ocular monitoring after the treatment is crucial to reducing the occurrence of ocular complications.

## Conclusions

Cisplatin contains platinum and has been linked to detrimental effects on ocular health. Although cisplatin-induced toxic optic neuropathy is rare, particularly in pediatrics, it can cause severe and irreversible visual impairment, even at therapeutic doses. Therefore, caution is necessary when cisplatin is involved in the treatment, and patients should be informed about the possible risks to their sensory organs and the potential for ocular complications. Early screening for ocular side effects and complications is imperative for patients after starting platinum-based chemotherapy.
